# A convenient renewable surface plasmon resonance chip for relative quantification of genetically modified soybean in food and feed

**DOI:** 10.1371/journal.pone.0229659

**Published:** 2020-02-26

**Authors:** Alexandra Plácido, Frederico Ferreira-da-Silva, José Roberto S. A. Leite, Noemí de-los-Santos-Álvarez, Cristina Delerue-Matos

**Affiliations:** 1 REQUIMTE/LAQV, Instituto Superior de Engenharia do Porto, Instituto Politécnico do Porto, Porto, Portugal; 2 Instituto de Investigação e Inovação em Saúde, i3S, Universidade do Porto, Porto, Portugal; 3 Instituto de Biologia Molecular e Celular, Universidade do Porto, Porto, Portugal; 4 Área Morfologia, Faculdade de Medicina, Campus Darcy Ribeiro, Universidade de Brasília, Brasília, Federal District, Brazil; 5 Departamento de Química Física y Analítica, Universidad de Oviedo, Oviedo, Spain; Huazhong University of Science and Technology, CHINA

## Abstract

The cultivation of genetically modified organisms (GMO) continues to expand worldwide. Still, many consumers express concerns about the use of GMO in food or feed, and many countries have legislated on labelling systems to indicate the presence of GMO in commercial products. To deal with the increased number of GMO events and to address related regulations, alternative detection methods for GMO inspection are required. In this work, a genosensor based on Surface Plasmon Resonance under continuous flow was developed for the detection and quantification of a genetically modified soybean (event GTS 40-3-2). In a single chip, the simultaneous detection of the event-specific and the taxon-specific samples were achieved, whose detection limits were 20 pM and 16 pM, respectively. The reproducibility was 1.4%, which supports the use of the chip as a reliable and cost-effective alternative to other DNA-based techniques. The results indicate that the proposed method is a versatile tool for GMO quantification in food and feed samples.

## Introduction

Genetically modified organisms (GMOs) are plants, animals, or microorganisms whose genetic composition had been altered by genetic engineering methods through the insertion of a new gene or by deletion of an existing one, in order to express a desired characteristic [1]. These modifications have contributed to the sustainable increase in productivity and to enhancing the chemical profile and nutritional quality of the derived products [2]. These characteristics have facilitated the establishment of genetically modified (GM) crops as the prevailing agricultural food products worldwide. Since the beginning of GM crops agriculture, there has been a 110-fold increase in the global use of biotech crops (185.1 million hectares in 2016), and 50% of this area corresponds to GM soybean [3]. The soybean GTS 40-3-2 event was developed to allow the use of glyphosate, an herbicide, as a weed control option [4]. This GM soybean variety contains the enzyme 5-enolpyruvylshikimate-3-phosphate synthase (EPSPS) isolated from the common soil bacterium, *Agrobacterium tumefaciens* strain CP4 (CP4 EPSPS), which confers tolerance to glyphosate [5].

The rapid growth of the GM crop industry has created controversies worldwide, and these in turn have fuelled the implementation of effective regulations to control their use [6]. The European Union (EU) has established one of the strictest legislations on GMO authorization and labelling. Reflecting concern about the presence of GMOs in food chain, the labelling is mandatory when the content of GMOs exceeds 0.9% of the ingredients. Below this threshold, when the presence of GMOs is accidental or technically unavoidable, labelling is waived [7]. It is therefore essential to develop detection strategies that are robust and allow detection and percentage quantification of GMOs.

Polymerase chain reaction (PCR) is the most commonly accepted method for identification and quantification of GMOs in feed and food samples, because of its versatility, sensitivity, specificity, and high-throughput applications [8,9]. However, it is laborious and expensive, which encourages researchers to develop new methodologies suitable for routine and rapid analysis. Great progress has been achieved by means of DNA-based testing sensors due to their simplicity, quickness, and reliability. A typical DNA biosensor is based on the recognition of hybridization events between an amplified target DNA sequence and complementary probes on transducers that convert the hybridization event into electrical or optical signals.

Surface plasmon resonance (SPR) is an optical technique useful for studying biomolecular interactions, and it occurs in the evanescent field generated by a thin gold-coated prism in contact with the analyte solution that flows through it [10]. SPR biosensors can be used to monitor DNA-DNA [11,12], protein-protein [13], protein-DNA [14], ligand-aptamer [15], enzyme-substrate or inhibitor [16], receptor-drug [17], lipid membrane-protein [18], protein-polysaccharide [19], and cell or virus-protein [20] interactions. In particular, DNA-based SPR biosensors are characterized by the interaction between a single-stranded oligonucleotide DNA (ssDNA) probe immobilized on the surface of a sensor chip, and the target DNA in solution via hybridization, resulting in an increase in the refractive index at the SPR sensor-solution interface [21]. For GMO analysis, the main SPR approaches are based on chemisorption of DNA probes onto Au chip surfaces [21,22] and/or on affinity interactions between streptavidin (SA) and biotinylated probes [17,23–25].

In the present work, a novel label-free system able to quantify GMO on the picomolar scale through the reversible capture of biotinylated DNA is reported. The platform uses specific biotinylated ssDNA capture probes coupled to a SPR sensor chip via SA-bound universal ssDNA, hybridized to the complementary strand anchored to the dextran matrix, which are able to detect Roundup Ready (RR) and lectin (Lec) target DNA with high sensitivity. This system can be completely regenerated and reused repeatedly, reducing the cost and time of analysis. For this GMO quantitative strategy, two assays were designed, one targeting an event-specific sequence of RR soybean, and the other the endogenous *lectin* gene, using the same sensor chip. The regenerative capacity of the chip surface was determinant in quantifying for the first time the presence of GMO by this methodology. Furthermore, it is noteworthy that the amplicons did not require any treatment before the injection, and that the proposed methodology was successfully applied to feed and food samples. Kinetic, conformational and thermodynamic analyses of the biomolecular interaction process were also studied.

## Materials and methods

### Reagents

4-(2-hydroxyethyl)piperazine-1-ethanesulfonic acid (HEPES, ≥99.5%), ethylenediaminetetraacetic acid (EDTA, ≥99.4%) concentrated saline sodium phosphate-EDTA (20× SSPE, pH 7.4) and DNA from herring sperm (D7290) were purchased from Sigma-Aldrich (St. Louis, MO, USA). Sodium chloride (NaCl, 99.5%) was supplied by PanReac AppliChem (Barcelona, Spain). Biotin CAPture Reagent, regeneration stock 1 and 2 (Biotin CAPture kit reagents) and surfactant P20 were obtained from GE Healthcare (Chicago, IL, USA). The buffers used were: (i) running buffer: HBS-EP (0.01 M HEPES pH 7.4, 0.15 M NaCl, 3 mM EDTA, 0.005% (v/v) surfactant P20); and (ii) hybridization buffer: SSPE (2× SSPE, pH 7.4). The DNA oligonucleotides (probes and target sequences) used in this work were purchased from NZYTech (Lisbon, Portugal), and the primers were synthesized by STAB Vida (Caparica, Portugal) as desalted products. Their sequences are listed in S1 Table. All stock solutions were prepared in Milli-Q water (specific resistivity 18.2 MΩ cm) and stored at -20°C.

### Biosensor construction and assay

**Fig 1:**
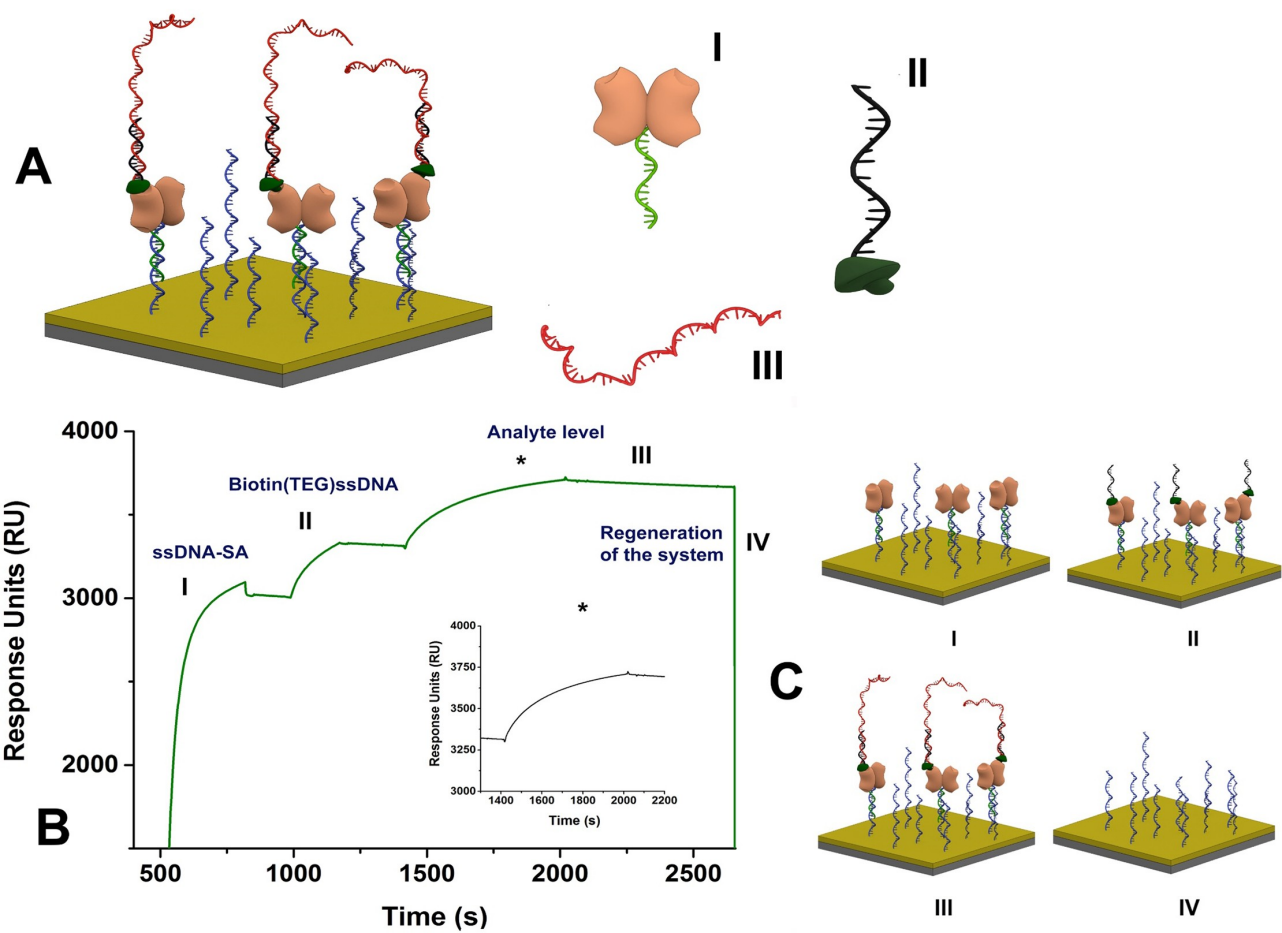
(**A**) Nomenclature of the DNA sequences used in the SPR-based biosensor construction: (**A.I**) ssDNA-streptavidin (ssDNA-SA); (**A.II**) ssDNA (black) linked to (TEG)Biotin (dark green); (**A.III**) RR or Lec target sequence (red). (**B**) Representative sensorgram of the novel capture concept used in this study to detect RR event. (**C**) Schematic illustration of the four steps that occurs in the SPR sensing surface (**C.I**) Hybridization of the ssDNA-SA with the complementary ssDNA (blue) linked on the chip surface; (**C.II**) Conjugation with biotinylated DNA probe by electrostatic interaction; (**C.III**) Hybridization between the analyte and capture probe; (**C.IV**) Regeneration.

The Biotin CAPture Kit was used for the interaction studies according to the manufacturer’s instructions with minor modifications. The surface of the CAP sensor consists of a carboxymethylated dextran matrix modified with a pre-immobilized universal ssDNA molecule. First, for surface conditioning and chip stabilization, three one-minute injections of regeneration solution and, subsequently, three complete start-up cycles were performed. Afterwards, 50 μg/mL of the biotin reagent CAP (ssDNA-SA) were injected at a flow rate of 5 μL/min for 5 min to build up the renewable SA-modified sensor chip to capture the biotinylated probe. Then the specific capture probe with a (TEG)Biotin spacer (TEG = triethylene glycol) prepared in HBS-EP buffer was injected at 30 μL/min for 3 min, to form a SA-biotin complex. Afterwards, the target was injected at 30 μL/min for 2 min. Finally, the regeneration of the surface after each analysis cycle was achieved by alkaline denaturation using the regeneration stock solutions at 30 μL/min for 2 min (Fig 1). Rehydration of the CAP sensor chip was performed by soaking it in HBS-EP buffer at room temperature overnight.

### Single-cycle kinetics experiments

Single-cycle kinetics (SCK) [26] experiments were used to characterize the kinetics of the hybridization events that constitutes the molecular basis of the detection strategy [27]. In this approach, several successive increasing concentrations of the analyte are injected, and only one regeneration step is performed at the end of the complete binding cycle in place of several cycles of binding and regeneration as in classical SPR experiments. Briefly, binding kinetics of a surface-based hybridization assay was carried out on the sensor chip after capturing low amounts of biotinylated ssDNA molecules (less than 400 RU) to avoid artefacts sometimes observed with high probe densities. These experiments were performed at a flow rate of 30 μL/min at 20°C [28], and the concentrations of the DNA target used were 0, 0.04, 0.2, 1, 5, and 10 μM. The injections of the analyte were carried out in duplicate.

### Conformational and thermodynamics studies

Circular dichroism (CD) spectroscopy analysis was performed in SSPE buffer at 60 μM. For all double-stranded DNA (dsDNA) used in the experiments, two ssDNA strands were hybridized in solution according to a previously described method [29]. Briefly, an equimolar concentration of each ssDNA, mixed in SSPE buffer, was subjected to a thermal shock (5 min at 98°C and 5 min in an ice bath) to facilitate the hybridization, and then the mixture was incubated for 25 min at room temperature. The spectra were converted to half molar ellipticity residue by using the relationship: [*θ*]/*θ* (10×c×n×d), where [*θ*] is the molar ellipticity (in degrees×cm^2^×dmol^-1^), *θ* the ellipticity in milli degrees, n is the number of peptide bonds, c is the molar concentration, d is the length of the cell in cm.

High resolution melt (HRM) analysis used SYTO^®^ as the intercalating dye (Invitrogen, Carlsbad, CA, USA), and the thermodynamic parameters were calculated according to the relationship between T_m_ and ΔG [30].

### Analytical performance analysis

The technique of multi-cycle analysis was used for the optimization of the analytical performance of the system. After hybridization of the Biotin CAPture Reagent to the basic sensor surface according to the manufacturer’s instructions, 50 μM of capture ssDNA-(TEG)Biotin in running buffer was injected at a flow rate of 30 μL/min for immobilization in the sample channel, leaving the reference channel unaltered. Immobilization levels of 1,000–1,200 RU were obtained over the injection time. Reduced levels were applied to specific experiments as indicated in results and discussion section. The analyte was serially diluted in running buffer to a concentration range of 0.1–8 nM and injected at 20°C at a flow rate of 30 μL/min for 300 s (association phase), followed by a dissociation phase of 1,200 s. The analyte or sample injection was performed in triplicate at different time points within each experiment. All data was double-referenced [31] using BiaEval 4.1 (Biacore^TM^).

### Sample preparation

Nine samples, including feeds, soybean bagasse, soybean seeds, soybean extract, textured soybean, and soy protein, were acquired in local supermarkets in 2016. They were triturated and homogenized using different blender containers previously treated with DNA decontaminator solution. Genomic DNA was extracted using the NucleoSpin food kit (Macherey-Nagel, Düren, Germany) according to the manufacturer’s instructions. The extracted DNA samples were stored at -20°C until further analysis. The purity and quantity of the extracted DNA were determined spectrophotometrically using NanoDrop^®^ ND-1000 (Thermo Fisher, Waltham, MA, USA) according to the manufacturer’s protocol. The oligonucleotide primer pairs Lec-F/Lec-R and RRS-Fm/RRS-Rm, targeting taxon-specific (lectin) and event-specific (RR) sequences, respectively, were used for amplification in end-point PCR. The amplicons were detected by SPR (S1 Table). The PCR amplifications were carried out in 25 μL of total reaction volume containing 2 μL of template DNA (100 ng/μL), 1× PCR buffer, 2 mM MgCl_2_, 200 μM dNTPs, 1 U Taq DNA Polymerase (recombinant), and 480 nM or 280 nM of each set of primers Lec-F/Lec-R and RRS-Fm/RRS-Rm, respectively. The reactions were performed in T100 thermal cycler PCR (BioRad, Hercules, CA, USA), using the following temperature programs: 95°C for 5 min; 40 cycles of 95°C for 30 s, 66 or 60°C for 30 s for primers Lec-F/Lec-R or RRS-Fm/RRS-Rm, respectively, and 72°C for 30 s; and a final extension at 72°C for 5 min. The amplified fragments were analyzed by electrophoresis in a 2% (w/v) agarose gel carried out in 1× TAE buffer (Tris acetic acid with EDTA) for 20 min at 190 V, stained with 1× Greensafe premium (NZYTech®, Lisbon, Portugal). The agarose gel was visualized under UV light tray Gel Doc^TM^ EZ System (BioRad, Hercules, CA, USA) and the digital image was obtained with Image Lab software version 5.1 (BioRad, Hercules, CA, USA).

For the SPR assays, the amplicons of taxon- and event-specific DNA from real samples were added without purification and after 1:11 and 1:1 dilutions in HBS-EP buffer, respectively.

### Instrumentation

All SPR experiments were carried out using a Biacore X100 (Biacore^™^, Uppsala, Sweden) analytical system, and a Biotin CAPture Kit (GE Healthcare Europe, Germany) for the interaction studies. Data were processed using BIAevaluation software 4.1 version (GE Healthcare Europe) and OriginPro 9.0 (OriginLab, Northampton, MA, USA) was used for equilibrium model analysis.

CD measurements were performed using a Jasco-815 spectropolarimeter (Jasco, Tokyo, Japan). The measurements were performed under nitrogen gas flow of 8 L/h at 25°C temperature, controlled by a Peltier system (JASCO). Spectra were acquired in a 0.3 cm path length quartz cuvette. Three scans were averaged per spectrum, operating from 200 to 320 nm at a scan speed of 20 nm/min and a bandwidth of 1 nm with data integration time 1–2 seconds, having subtracted the buffer contribution and normalized for concentration.

HRM analysis and T_m_ determination among ssDNA used in this study were performed on the Rotor-Gene 6000^™^ (Corbett Research, Mortlake, Australia), from 45 to 95°C, with a temperature increase at the rate of 0.2°C/s for all assays, in triplicate.

## Results and discussion

### Biosensor design

With the objective of developing a sensitive platform for GMO quantification, a novel SPR-based biosensor was designed targeting the transgenic construct of Roundup Ready (RR) soybean and the taxon-specific soybean gene, *lectin*. For this purpose, the Sensor Chip CAP was used as an alternative for the first SPR DNA-based sensors [17], which used SA attached directly to the surface of the chip. In Fig 1, schematic drawings depict the immobilization strategy. SA conjugated with a universal ssDNA molecule is bound to a complementary sequence immobilized on the surface of the Sensor Chip CAP by oligo hybridization (Fig 1C, step I). The biotinylated capture probe is captured by the system (Fig 1C, step II), and then the interaction with the analyte is studied (Fig 1C, step III). Lastly, the surface is regenerated and rebuilt in the next cycle (Fig 1C, step IV). The same chip was used in the assays with the taxon- and event-specific sequences.

Fig 1B shows a representative sensorgram obtained during multi-cycle analysis for RR event. In step I, hybridization of the reagent (ssDNA-SA) with the DNA covalently attached to the chip was very efficient, resulting in over 3,000 RU for both targets (RR and Lec), which is determinant for the sensitivity of the biosensor. For most proteins, binding of ~ 1 ng/mm^2^ of protein at the dextran surface causes a signal change of 1,000 RU [32], though the exact relationship varies with the refractive index of the bound molecule. However, the total surface area is mostly unknown and has a thickness ranging from 20–50 nm of dextran, becoming rough. In our case, as the initial surface is covered with dextran and ssDNA, this analysis is an estimation. The ssDNA-SA complex has approximately 60 kDa, equivalent to 3.01 × 10^10^ molecules/mm^2^. Considering that only two out of four binding sites are available for biotin binding, there are 6.02 × 10^10^ sites/mm^2^. The injection of ssDNA-(TEG)Biotin (capture probe) gave approximately 3,400 RU (step II) for both systems (RR and Lec), which is expected since both capture probes have the same size (25-mer). The maximum RU theoretical value can be calculated by using the following expression [RU_max_ = molecular mass (analyte/capture probe) × RU_capture probe_] [33], which gives, for instance, 812 RU for RR capture probe. This results in a coating of about 50%, leaving in theory ~ 3 × 10^10^ molecules/mm^2^ for target detection. Full coverage is not expected due to the size and charge of the biotinylated molecule. The capture probe was designed with a TEG spacer group to increase the oligo-biotin distance by 15 atoms [34, 35], avoiding steric hindrance. This strategy has already been successfully applied in the development of several nanodevices [36–38]. In step III, the addition of the analyte increases the analytical signal due to the hybridization with the capture probe. Then, the dissociation occurs at a very slow rate as indicated by the very small slope of the signal. The surface regeneration (step IV) occurs by dsDNA denaturation. Thus, the chip surface returns to its initial condition, becoming ready for the next cycle. After around 20 regeneration steps, the sensor surface remains robust and its results are replicable, as it can be seen by the relative standard deviation (RSD) of the inter-day reproducibility at 8 nM that was 0.4% and 0.5% for RR event and lectin, respectively. Furthermore, the intra-day reproducibility at 8 nM was also evaluated for both targets and the outcomes of RSD were 0.2% (RR) and 0.3% (lectin), indicating a very good precision for the biosensor.

### Kinetics characterization

**Fig 2:**
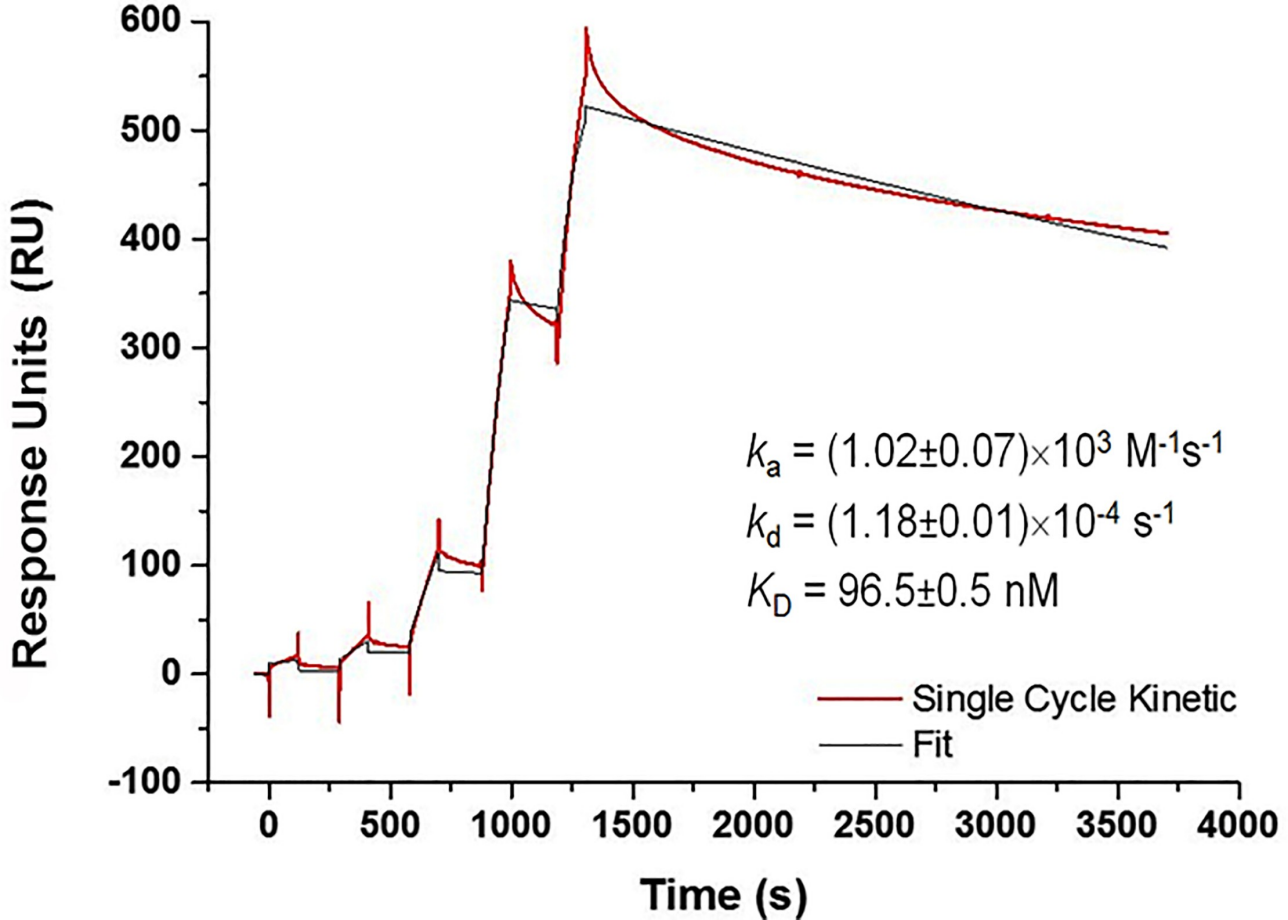
Sensorgram representative of the SCK analysis of the duplex DNA formation on the biosensor. The SPR experiments were performed at 20°C using 30 μL/min of flow rate and 50 μg/mL of capture probe. The red curve represents the experimental data and the black curve represents the fit of the sensorgram to a Langmuir 1:1 model of interaction. χ^2^ ranged from 84 to 115 (RU^2^), and the U-Value was 1. *k*_a_ and *k*_d_ are the association and dissociation rate constants, respectively, and *K*_D_ is the dissociation equilibrium constant. The experiments were performed in triplicate (n = 3).

The biosensor model developed in this work was used to investigate complexes dissociating from the functionalized chip surface by SCK. The association and dissociation rate constants (*k*_a_ and *k*_d_, respectively), were determined by direct curve fitting of the sensorgrams to an interaction model of Langmuir 1:1 [26]. The dissociation equilibrium constant (*K*_D_) of the hybridization was calculated as *k*_*d*_*/k*_*a*_. The obtained values are shown in Fig 2 and reveal a very rapid association and very slow dissociation. The equation for fitting the sensorgrams was assumed as the algebraic sum of two equations: 1) the equation for fitting a data set of five concentrations of DNA target injected sequentially; and 2) the equation for fitting the dissociation phase. In SPR, to evaluate the reliability of the experimental data it is usual to apply the uniqueness value (U-Value) parameter for the kinetic rate constants, and the values below 15 are considered statistically optimal. The U-value obtained in all experiments (triplicate) was around 1, which shows the reliability of the results obtained. Studies with synthetic oligonucleotides (10 bp) and using a DNA probe attached to SA directly on the dextran surface estimated *k*_a_ and *k*_d_ around 10^5^ M^-1^ s^-1^ and 10^−3^ s^-1^, respectively [27]. It is not surprising that, in our work, *k*_a_ and *k*_d_ were slower (10^3^ M^-1^ s^-1^ and 10^−4^ s^-1^, respectively); since our target is longer (84 bp), the capture probe access becomes more difficult and the duplex more stable.

Statistical methods, such as the calculation of the U-value, are very important for kinetic validation, since parameters like DNA probe size, sequence, secondary structure, and density on the sensor surface influence the results. In our work, the value found of *K*_D_ was about 10^−7^ M, which is in accordance with the value obtained for shorter oligonucleotides (1–10 × 10^−8^ M range) [36, 39]. As mentioned above, the larger target size compared with capture probe size contributed to slowing down the dissociation process [40]. Regarding Lec DNA target sequences, the equilibrium constant (*K*_D_ = 6.9 × 10^−7^ nM) was determined using the equilibrium model [15], as shown in S1 Fig, because the even slower dissociation rate yielded unreliable fitting to the kinetic model.

### DNA conformational and thermodynamics studies

**Fig 3:**
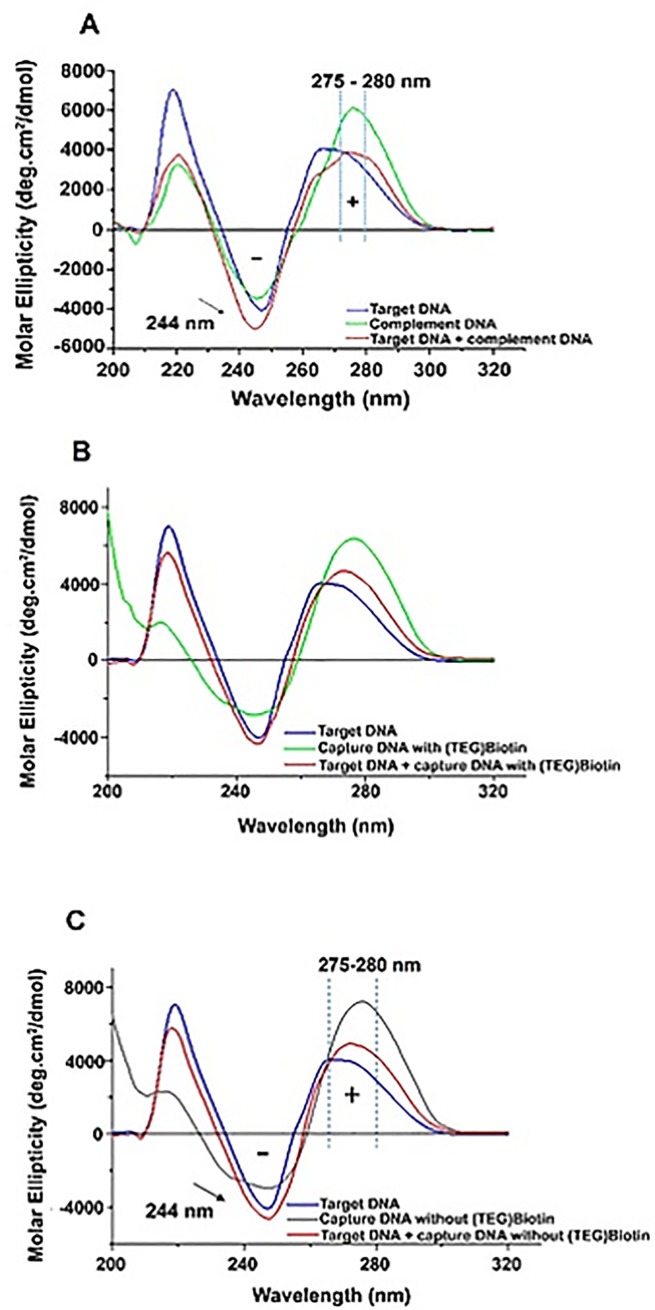
CD spectra of (**A**) target ssDNA, complement ssDNA and the corresponding duplex; target ssDNA and capture DNA with (**B**) and without (**C**) the (TEG)Biotin group and the corresponding duplexes. All spectra were acquired in a 0.1 cm path length quartz cuvette at 25°C, and RR DNA sequences were analyzed at 60 μM in 2× SSPE buffer (pH 7.4).

The conformational changes in the secondary structure of ssDNA and dsDNA, from capture probe and target of the RR event, were studied using CD spectroscopy (Fig 3). CD is a very sensitive and accurate technique based on chiral molecules interactions with circularly polarized electromagnetic rays, representing the difference of the absorption of right- and left-handed circularly polarized light by chiral molecules, such as DNA [41,42]. Thus, CD spectroscopy is primarily used in studies of DNA, presenting many advantages over other methods of conformational analysis. On the other hand, its high sensitivity enables work with small quantities of DNA (*e*.*g*., less than 25 μg) [41,43] and can easily discriminate between several secondary structures formed by DNA (A-, B-, and Z-), since all the forms of DNA present a unique CD spectrum with different maximum wavelength and ellipticity [44,45].

As can be seen in Fig 3, the CD spectra of all ssDNA and dsDNA consisted of a positive peak at 275 nm and a negative peak at 244 nm, which is characteristic of the B-form of DNA, the conformation most commonly observed. Changes in the signal at 275 nm could be observed in all three systems analysed. Because B-DNA is stable, a DNA probe design that stabilizes in this form can be advantageous for application in biosensors, since it leads to a decrease in Gibbs energy and, thus, increases the association constant between capture DNA and its associated target. Fig 3A shows the behavior of the target ssDNA, complement ssDNA, and its hybridized form. CD data corroborate that, the B-DNA form is evident according to both the 275–280 and 244 nm signals. In addition, the signal intensity at 244 nm and its left shift indicate a still higher proportion of B-DNA form after hybridization (Fig 3A and 3B), which may be related to the longest complementarity (right-handed). However, as can be seen in Fig 3B, CD data for dsDNA shows a left shift at 244 nm relative to ssDNA, which does not occur when compared to the ssDNA and dsDNA shown in Fig 3C (absence of (TEG)Biotin). These data suggest a higher stability after the hybridization of the (TEG)Biotin system, which makes the system relevant for the development of biosensors, as it is directly related to target DNA detection.

**Fig 4:**
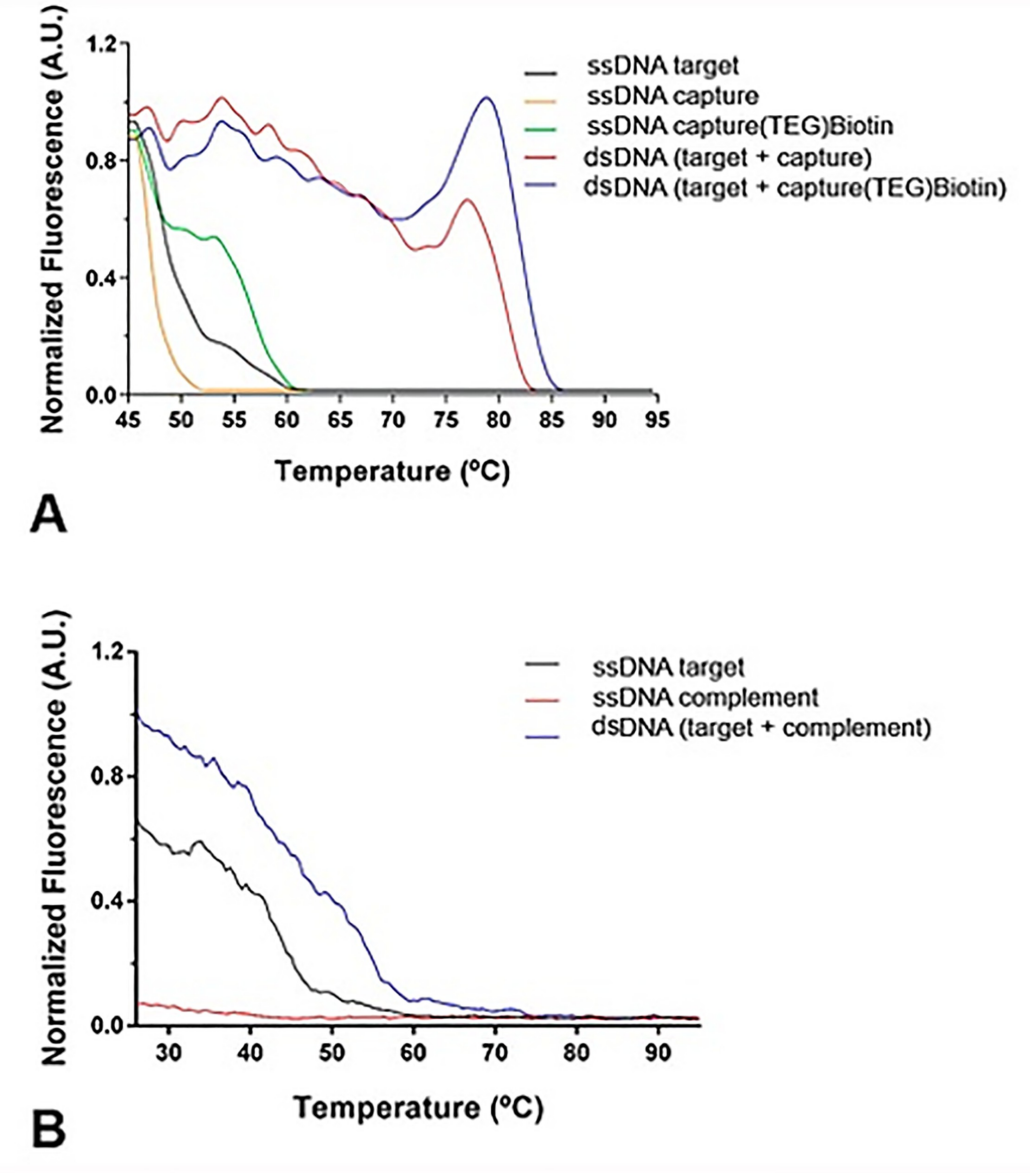
High resolution melt curve. (**A**) dsDNA(Target+capture): T_m_ = 78 ᵒC; dsDNA(target+capture-(TEG)Biotin): T_m_ = 82 ᵒC; (**B**) and dsDNA(target+complement): T_m_ = 53 ᵒC. All analyzes are compared to the corresponding ssDNA (ssDNA target or complement).

Fig 4 shows the sharp decrease in fluorescence that occurs when the dsDNA melts into its single stranded form (ssDNA), during the denaturation process. These curves allow T_m_ estimation since a direct relationship with thermodynamic parameters exists. For the three types of interactions studied, the results were the following: dsDNA(target + capture): T_m_ = 78°C/ΔG = −45.03 kJ/mol; dsDNA(target + (TEG)Biotin capture): T_m_ = 82°C/ΔG = −45.55 kJ/mol; and dsDNA(target + complement): T_m_ = 53°C/ΔG = −41.83 kJ/mol. The results show that the hybridization of target ssDNA and capture ssDNA-(TEG)Biotin is slightly more stable than the hybridization without the (TEG)Biotin group (ΔG = −45.03 kJ/mol), which is in accordance with CD data that show there are few conformational differences. The highest T_m_ value was obtained for dsDNA(target + (TEG)Biotin capture) showing that the presence of (TEG)Biotin hinders the separation of DNA strands (Fig 4A). Comparatively, the hybridization of target ssDNA with the complement ssDNA results in a smaller T_m_, probably because the composition and size of the oligonucleotides of both ssDNA can lead to a steric hindrance (Fig 4B).

The HRM technique for determination of T_m_ is able to detect small differences in the thermodynamics of the process caused by duplex DNA. The correlation between conformational and thermodynamic methods allows researchers to better understand the system and consequently might even direct the development of DNA-based biosensors.

### Analytical performance

**Fig 5:**
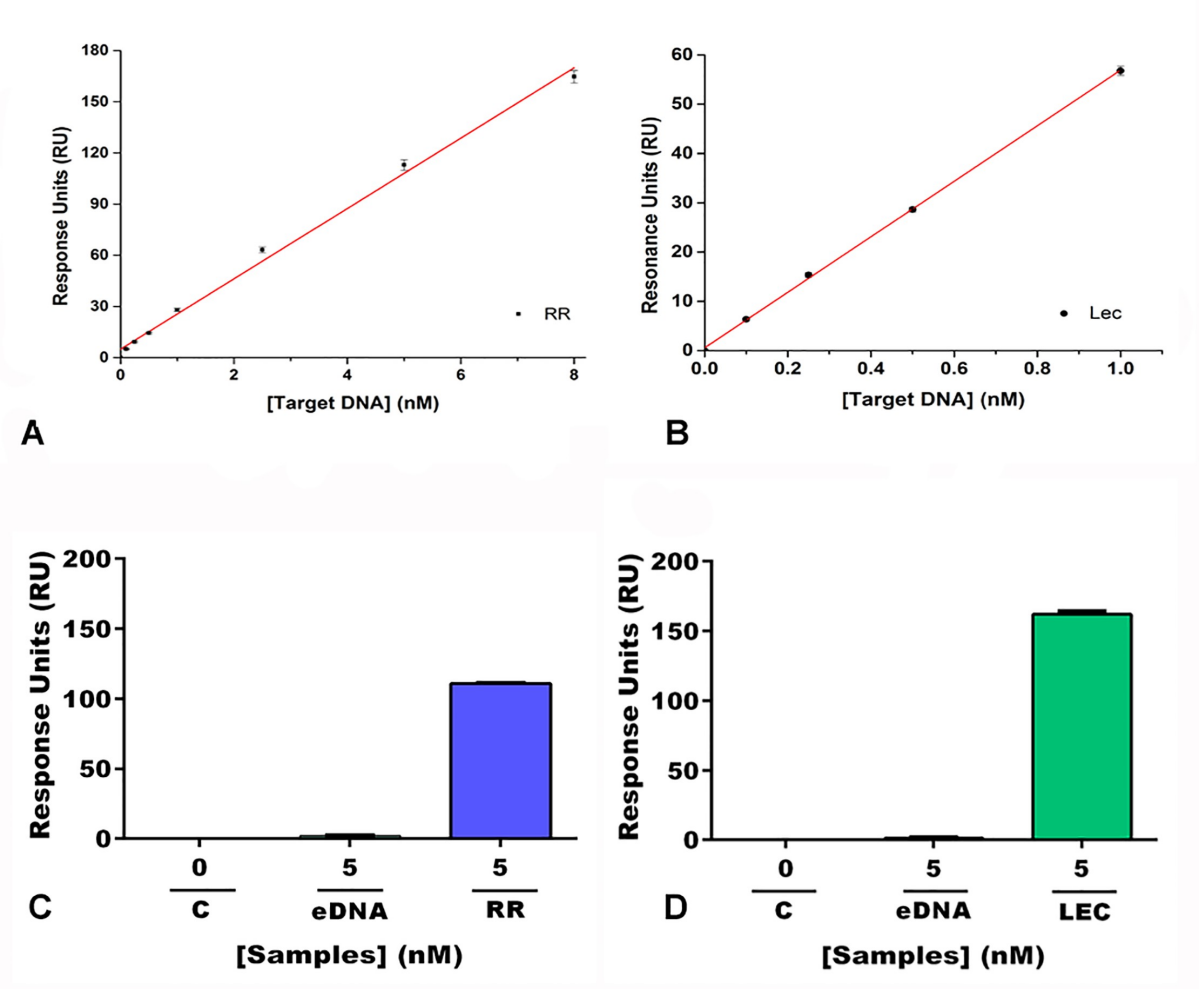
Calibration plots for the (**A**) event-specific and (**B**) reference targets. RU values obtained for blank experiment, exogenous DNA (herring sperm), and target (**C**) RR and (**D**) Lec DNA sequences at 5 nM.

The calibration plots for RR and Lec obtained from optimized multi-cycle analysis at different concentrations (0 to 8 nM) are shown in Fig 5. A linear range from 0.1 to 8 nM and 0.1 to 1 nM was achieved for RR and Lec targets, respectively, according to the following equations: RU = 20.6 (±0.5) [RR] (nM) + 4.8 (±1.8) (r = 0.9982, n = 3) (Fig 5A); RU = 56.3 (±0.7) [Lec] (nM) + 0.6 (±0.3) (r = 0.9998, n = 3) (Fig 5B). The limits of detection(LOD) and limits of quantification (LOQ) were calculated as 3 s_y/x_/m and 10 s_y/x_/m, respectively, where s_y/x_ is the standard deviation of the regression and m is the slope of the calibration plot. The LOD values were 20 pM and 16 pM for RR and Lec, respectively, and the LOQ were 70 pM (RR) and 53 pM (Lec). LOD and LOQ in the picomolar range are within the lowest label-free optical systems, and these results show the sensitivity of this new surface biosensor design. The method is more sensitive to lectin than to RR, probably because of the differences between the secondary structures of the capture probes, namely loop location and size. As can be seen in S2 Fig, the Lec capture probe forms a small 11-nt-loop at the 3´end (S2B Fig), which causes lesser steric hindrance than the 16-nt-loop at the 5´end of RR capture probe (S2A Fig). This result is in good agreement with the higher slope for lectin also found in a recently reported electrochemical device using identical capture probes [29].

The ability of SPR-based detection of nucleic acids (NA) at nanomolar or high subnanomolar range depends not only on the SPR instrument’s sensitivity but also on the hybridization conditions, mainly ionic strength and target size. Most hybridization-based SPR sensors present a LOD ranging from 0.1 to 10 nM [46], and only one work, based on the hybridization event between the unlabeled DNA and complementary thiolated-DNA, managed to reach a LOD of 20 pM [47]. In our case we speculate that we can also benefit from an optimum DNA strand distribution due to the immobilization strategy chosen that precludes the formation of islands of highly packed DNA strands where hybridization is hindered (an unavoidable problem with thiolated DNA on Au surfaces) [48] but provides high enough strands to obtain significant signal changes at low target concentrations.

In the field of electrochemical transduction, the use of magnetic (nano/micro)particles as a platform for the genoassays reached LOD ranging from 20/50 pM [29] to 0.9/0.3 pM [49] for RR/Lec. In both studies, the hybridization was performed in a sandwich format using a signaling probe labelled with fluorescein isothiocyanate (FITC), followed by enzymatic labelling using a Fab fragment anti-FITC conjugated to peroxidase. Comparatively, our study, a label-free platform, is able to achieve LOD at a low picomolar range and allows easy automation to analyze hundreds of samples on a large lab routine scale, similar to known systems for drug discovery.

The reproducibility of the biosensor at 5 nM of RR and Lec was analyzed under the optimized conditions. The relative standard deviation (RSD) of the intra-day measurements were 0.1% (RR) and 0.3% (Lec), while the inter-day reproducibility was 1.4% (RR) and 0.5% (Lec), indicating a very good precision for both assays.

The selectivity was investigated using a non-complementary sequence corresponding to 5 nM of an exogenous DNA (eDNA) from herring sperm. The experiments resulted in minimal detection, near to a blank response, confirming the specificity of the assays (Fig 5C and 5D).

In order to improve the analytical performance, a sandwich assay using a third DNA sequence, fully complementary to the remaining unhybridized target, was performed for the RR event (S3 Fig). The regression equation was: RU = 25 (±1) [ds DNA(target+complement)] (nM) + 18.1 (±0.5) (r = 0.996, n = 3) with a LOD and LOQ of 11 and 36 pM, respectively. Since the mass increment strategy provided only a limited improvement in LOD, this approach was not used in further experiments.

### Quantitative food and feed sample analysis

**Fig 6:**
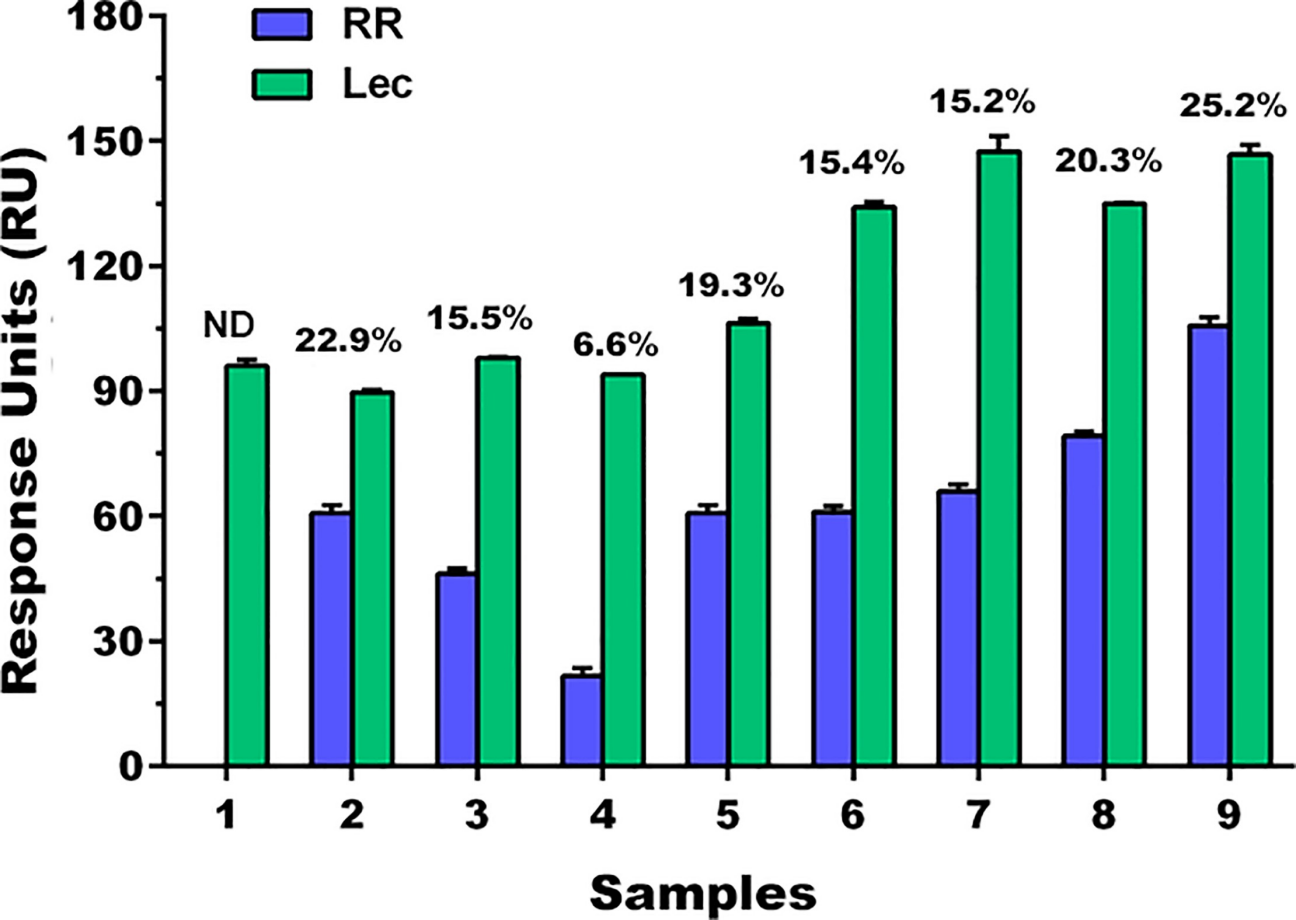
Analytical responses obtained after dilution of event-specific RR (1:1) and Lec (1:11) amplified DNA of nine real samples. The quantitative results were expressed as RR percentages determined by RR/LEC. ND: Not Detected.

The usefulness of the developed method to quantify the GMO percentage was verified in real food and feed samples. First, the DNA was extracted and its quality and purity were determined. After checking the presence of amplifiable DNA, all samples were subjected to taxon-specific PCR and, if positive, to RR PCR amplification. All of them were positive for both amplifications (S4 Fig). In the developed SPR assays, we used the amplicons without purification in substitution of the synthetic oligonucleotide targets. According to the legislation, the GMO percentage is determined by the ratio between the value of the GM soybean event (RR) measurement and the value of the endogenous gene (lectin), in ng, using the regression equations and the molar mass of each sequence. RR was not quantifiable in one of the samples because the signal was below the one for the LOQ. In the rest of the samples, RR content ranges from 6.6 to 25.2% (Fig 6), evidencing that the novel capture concept used in this work is a good alternative for quantification of a GTS 40-3-2 event in feed and food samples. It has been reported that disruption of amplicons by high temperature or denaturing agents is needed before SPR measurement [46]. Here we have shown that it can be carried out without any additional treatment.

## Conclusions

We report the first SPR biosensor developed for GMO quantification in feed and food samples according to the legislation guidelines that do not rely on qPCR or labels for detection. The use of hybridization reaction as a recognition step add extra selectivity as it has been previously demonstrated [50]. This approach requires two assays, one targeting the taxon-specific gene and the other targeting the event-specific sequence, using a single renewable sensor chip. The capture concept used in the study proved to be rapid, sensitive, and reproducible. Furthermore, the amplicons from real samples were applied directly without any purification and denaturation steps. The DNA duplex [dsDNA(target+capture(TEG)Biotin] was characterized kinetically, showing very rapid association (*k*_a_) and a very slow dissociation (*k*_d_) rates. The SCK is a fast method which is very promising for studies of this nature. Conformational and thermodynamic studies demonstrated that ssDNA and dsDNA predominantly adopt right-handed B-DNA and that the addition of anchor groups to the DNA probe, such as TEG, increases the stability of the interaction with target DNA. The platform presented shows great potential for the determination of the GMO percentage, since it provides a quick response and has a lower cost due to the regenerability of the sensor surface, an approach never previously explored for GMO quantification in food and feed. SPR instrumentation has advanced rapidly in the last years and now miniaturized portable devices exist that would allow performed the proposed assays in low resources settings in a decentralized manner. Besides there are multichannel instruments that allow parallel analysis of multiple samples shortening the analysis time and improving the throughput though the equipment is less economic.

## Supporting information

S1 File Amplification strategy protocol. (PDF)

S1 Table Oligonucleotide primer pairs Lec-F/Lec-R and RRS-Fm/RRS-Rm, targeting taxon-specific (lectin) and event-specific (RR) sequences used for amplification by end-point PCR and amplicons detected by SPR. (PDF)

S1 Fig Steady-state affinity (*K*_D_, equilibrium) determination between Lec capture probe and target DNA Lec at a flow rate of 30 μL/mL by titration of analyte. (PDF)

S2 Fig Secondary structures of (A) RR and (B) Lec capture probes at 25°C and 0.3 M Na^+^ from mfold web server.
(PDF)

S3 Fig Signal amplification strategy to improve the analytical performance of the RR system. (**A**) Schematic representation of the hybridization between the ligand (capture probe RR) and the analyte [dsDNA(target+complement)] on the chip surface; (**B**) Sensorgram from optimized multi-cycle analysis at different concentrations of target DNA (0 to 8 nM); (**C**) Calibration plot obtained in a concentration range 0–8 nM; (**D**) Schematic representation of the hybridization procedure in solution between target DNA and complement DNA; (**E**) Gel electrophoresis before and after hybridization between the target ssDNA and the complement ssDNA: (**E.I**) DNA ladder; (**E.II**) ssDNA target; (**E.III**) dsDNA(target+complement); (**E.IV**) ssDNA complement.
(PDF)

S4 Fig Agarose gel electrophoresis (2%) of PCR products with primers targeting (**A**) RR event-specific and (**B**) *lectin* gene in food samples. Lines SJ1-SJ9: soybean samples and M: 100 bp ladder.
(PDF)
