# Supplementary material for: A convenient renewable surface plasmon resonance chip for relative quantification of genetically modified soybean in food and feed
Source: PLoS One. 2020 Feb 26;15(2):e0229659. doi: 10.1371/journal.pone.0229659 (PMC7043770; doi:10.1371/journal.pone.0229659)
Supplement: S1 File — (PDF) [file pone.0229659.s001.pdf]

AMPLIFICATION STRATEGY PROTOCOL

A signal amplification strategy was tested using as a model a sandwich-type hybridization assay (Fig. S3, A). For that purpose, a 0.1 µM equimolar concentration of target ssDNA and complement ssDNA were mixed in 2× SSPE buffer (final volume of 500 µL) to study a concentration range of 0.1–8 nM (Fig. S3, B, C). Then, to promote the hybridization event, each vial tube was subjected to a thermal shock consisting of 5 min at 98 °C and 5 min in an ice bath, and lastly, the tubes were left for 25 min at room temperature (Fig. S3, D) [1]. Subsequently, 95 µL of the partial duplex DNA in solution was injected, allowing that the heterogeneous hybridization reaction occurs and there is the mass increase in the system.

To confirm the formation of dsDNA was carried out an electrophoresis gel before and after the hybridization between the target ssDNA and complement ssDNA (Fig. S3, E).

Reference

[1] A. Plácido, C. Pereira, A. Guedes, M.F. Barroso, R. Miranda-Castro, N. de-los-Santos-Álvarez, C. Delerue-Matos, Electrochemical genoassays on gold-coated magnetic nanoparticles to quantify genetically modified organisms (GMOs) in food and feed as GMO percentage, Biosens. Bioelectron. 110 (2018) 147-154.
