# Supplementary material for: A convenient renewable surface plasmon resonance chip for relative quantification of genetically modified soybean in food and feed
Source: PLoS One. 2020 Feb 26;15(2):e0229659. doi: 10.1371/journal.pone.0229659 (PMC7043770; doi:10.1371/journal.pone.0229659)
Supplement: S1 Table — (PDF) [file pone.0229659.s002.pdf]

S1 Table. oligonucleotide primer pairs Lec-F/Lec-R and RRS-Fm/RRS-Rm, targeting taxon-specific (lectin) and event-specific (RR) sequences used for amplification by end-point PCR and amplicons detected by SPR

	Target	Name/description	Sequence (5'→3')	Amplicon (bp)	Mw (Da)	Reference
SPR assay	*Lectin* gene	Taxon-specific target	CCAGCTTCGCCGCTTCCTTCAACTTCACCTTCTATGCCCCTGACACAAAAAGGCTTGCAGATGGGCTTGCCTTC	74	22494.6	[25]
		Capture probe	Biotin(TEG)AAGTTGAAGGAAGCGGCGAAGCTGG		8503.6	
	35S/Plant junction	Transgenic target	TTCATTCAAAATAAGATCATACATACAGGTTAAAATAAACATAGGGAACCCAAATGGAAAAGGAAGGTGGCTCCTACAAATGCC	84	25974	[25]
		Capture probe	Biotin(TEG)GGCATTTGTAGGAGCCACCTTCCTT		8194	
		Complement	TTCCATTTGGGTTCCCTATGTTTATTTTAACCTGTATGTATGATCTTATTTTGAATGAA		18679	
End-Point PCR	*Lectin* gene	Lec-F	CCA GCT TCG CCG CTT CCT TC	74	5955.9	[26]
		Lec-R	GAA GGC AAG CCC ATC TGC AAG CC		7027.6	
	35S/Plant junction	RRS-Fm	TTC ATT CAA AAT AAG ATC ATA CAT ACA GG	84	8876.9	[26]
		RRS-Rm	GGC ATT TGT AGG AGC CAC CTT C		6726.4	
