# Supplementary material for: A convenient renewable surface plasmon resonance chip for relative quantification of genetically modified soybean in food and feed
Source: PLoS One. 2020 Feb 26;15(2):e0229659. doi: 10.1371/journal.pone.0229659 (PMC7043770; doi:10.1371/journal.pone.0229659)
Supplement: S1 Fig — (PDF) [file pone.0229659.s003.pdf]

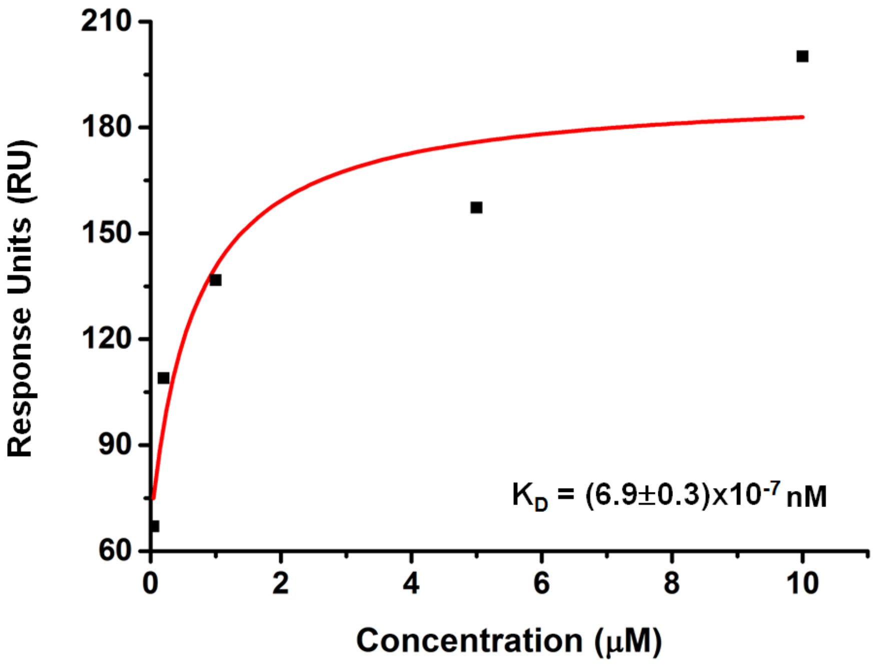

S1 Fig. Steady-state affinity (K_D, equilibrium) determination between Lec capture probe and target DNA Lec at a flow rate of 30 µL/mL by titration of analyte.
