# Supplementary material for: A convenient renewable surface plasmon resonance chip for relative quantification of genetically modified soybean in food and feed
Source: PLoS One. 2020 Feb 26;15(2):e0229659. doi: 10.1371/journal.pone.0229659 (PMC7043770; doi:10.1371/journal.pone.0229659)
Supplement: S2 Fig — Secondary structures of (A) RR and (B) Lec capture probes at 25°C and 0.3 M Na+ from mfold web server. (PDF) [file pone.0229659.s004.pdf]

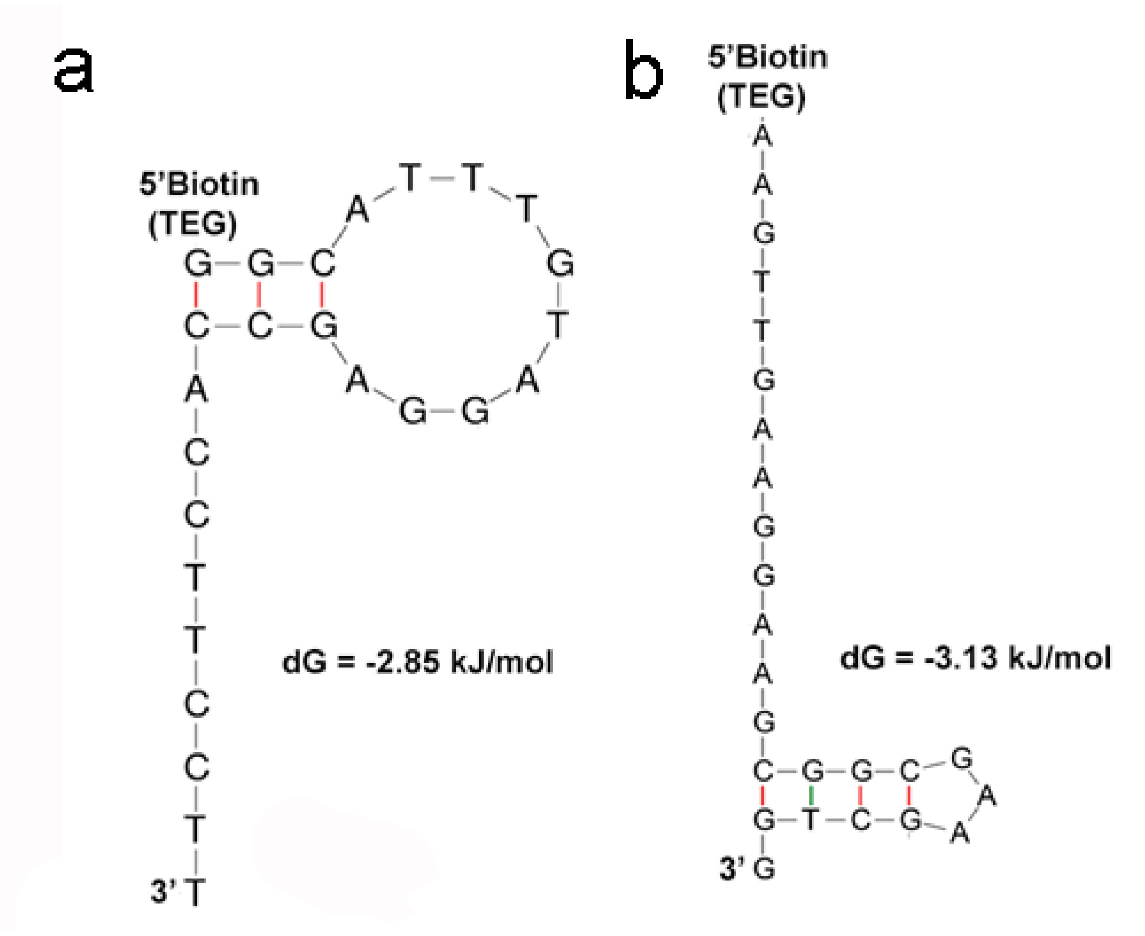

S2 Fig. Secondary structures of (A) RR and (B) Lec capture probes at 25ºC and 0.3 M Na^+ from mfold web server.
