# Supplementary material for: A convenient renewable surface plasmon resonance chip for relative quantification of genetically modified soybean in food and feed
Source: PLoS One. 2020 Feb 26;15(2):e0229659. doi: 10.1371/journal.pone.0229659 (PMC7043770; doi:10.1371/journal.pone.0229659)
Supplement: S3 Fig — (A) Schematic representation of the hybridization between the ligand (capture probe RR) and the analyte [dsDNA(target+complement)] on the chip surface; (B) Sensorgram from optimized multi-cycle analysis at different concentrations of target DNA (0 to 8 nM); (C) Calibration plot obtained in a concentration range 0–8 nM; (D) Schematic representation of the hybridization procedure in solution between target DNA and complement DNA; (E) Gel electrophoresis before and after hybridization between the target ssDNA and the complement ssDNA: (E.I) DNA ladder; (E.II) ssDNA target; (E.III) dsDNA(target+complement); (E.IV) ssDNA complement. (PDF) [file pone.0229659.s005.pdf]

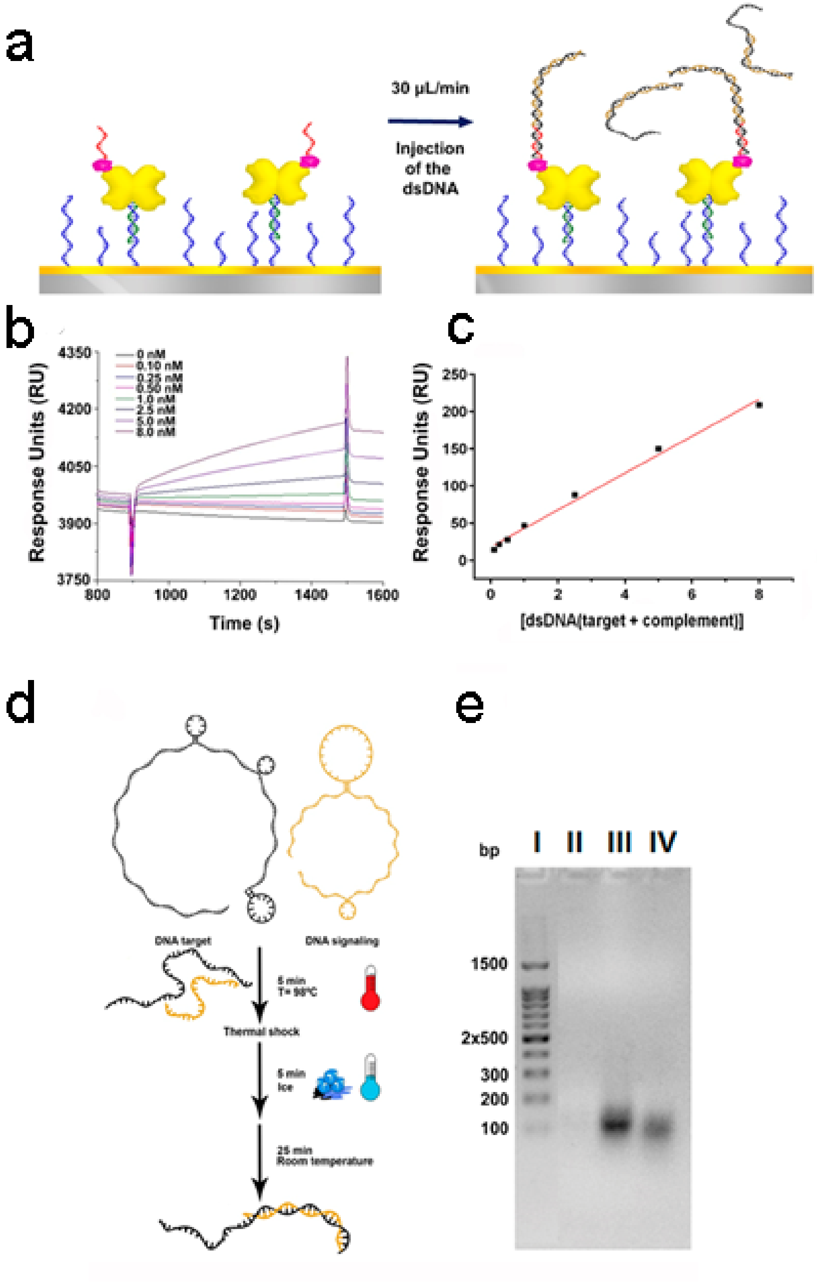

S3 Fig. Signal amplification strategy to improve the analytical performance of the RR system. (**A**) Schematic representation of the hybridization between the ligand (capture probe RR) and the analyte [dsDNA(target+complement)] on the chip surface; (**B**) Sensorgram from optimized multi-cycle analysis at different concentrations of target DNA (0 to 8 nM); (**C**) Calibration plot obtained in a concentration range 0-8 nM; (**D**) Schematic representation of the hybridization procedure in solution between target DNA and complement DNA; (**E**) Gel electrophoresis before and after hybridization between the target ssDNA and the complement ssDNA: (**E.I**) DNA ladder; (**E.II**) ssDNA target; (**E.III**) dsDNA(target+complement); (**E.IV**) ssDNA complement.
