# Supplementary material for: A convenient renewable surface plasmon resonance chip for relative quantification of genetically modified soybean in food and feed
Source: PLoS One. 2020 Feb 26;15(2):e0229659. doi: 10.1371/journal.pone.0229659 (PMC7043770; doi:10.1371/journal.pone.0229659)
Supplement: S4 Fig — Agarose gel electrophoresis (2%) of PCR products with primers targeting (A) RR event-specific and (B) lectin gene in food samples. Lines SJ1-SJ9: soybean samples and M: 100 bp ladder. (PDF) [file pone.0229659.s006.pdf]

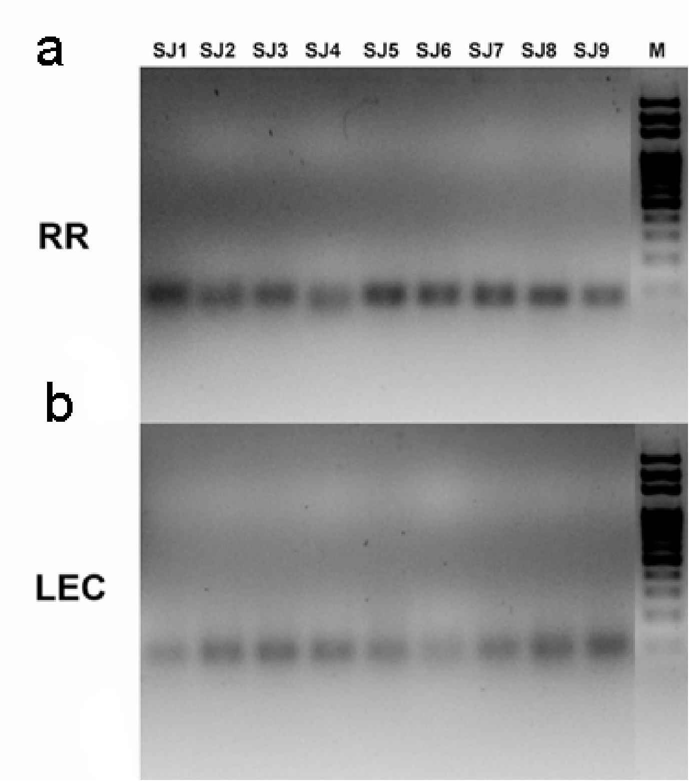

S4 Fig. Agarose gel electrophoresis (2%) of PCR products with primers targeting (**A**) RR event-specific and (**B**) *lectin* gene in food samples. Lines SJ1-SJ9: soybean samples and M: 100 bp ladder.
